# Using machine learning approach for screening metastatic biomarkers in colorectal cancer and predictive modeling with experimental validation

**DOI:** 10.1038/s41598-023-46633-8

**Published:** 2023-11-08

**Authors:** Amirhossein Ahmadieh-Yazdi, Ali Mahdavinezhad, Leili Tapak, Fatemeh Nouri, Amir Taherkhani, Saeid Afshar

**Affiliations:** 1 Research Center for Molecular Medicine, Hamadan University of Medical Sciences, Hamadan, Iran; 2 Department of Medical Biotechnology, School of Advanced Medical Sciences and Technologies, Hamadan University of Medical Sciences, Hamadan, Iran; 3 Department of Biostatistics, School of Public Health and Modeling of Noncommunicable Diseases Research Center, Hamadan University of Medical Sciences, Hamadan, Iran; 4 Department of Pharmaceutical Biotechnology, School of Pharmacy, Hamadan University of Medical Sciences, Hamadan, Iran; 5 Cancer Research Center, Hamadan University of Medical Sciences, Hamadan, Iran

**Keywords:** Cancer, Molecular biology, Systems biology

## Abstract

Colorectal cancer (CRC) liver metastasis accounts for the majority of fatalities associated with CRC. Early detection of metastasis is crucial for improving patient outcomes but can be delayed due to a lack of symptoms. In this research, we aimed to investigate CRC metastasis-related biomarkers by employing a machine learning (ML) approach and experimental validation. The gene expression profile of CRC patients with liver metastasis was obtained using the GSE41568 dataset, and the differentially expressed genes between primary and metastatic samples were screened. Subsequently, we carried out feature selection to identify the most relevant DEGs using LASSO and Penalized-SVM methods. DEGs commonly selected by these methods were selected for further analysis. Finally, the experimental validation was done through qRT-PCR. 11 genes were commonly selected by LASSO and P-SVM algorithms, among which seven had prognostic value in colorectal cancer. It was found that the expression of the *MMP3* gene decreases in stage IV of colorectal cancer compared to other stages (P value < 0.01). Also, the expression level of the *WNT11* gene was observed to increase significantly in this stage (P value < 0.001). It was also found that the expression of *WNT5a, TNFSF11*, and *MMP3* is significantly lower, and the expression level of *WNT11* is significantly higher in liver metastasis samples compared to primary tumors. In summary, this study has identified a set of potential biomarkers for CRC metastasis using ML algorithms. The findings of this research may provide new insights into identifying biomarkers for CRC metastasis and may potentially lay the groundwork for innovative therapeutic strategies for treatment of this disease.

## Introduction

Despite numerous research efforts aimed at identifying strategies for cancer prevention, colorectal cancer (CRC) is still the second cause of cancer mortality worldwide, with about one million deaths in 2020^1^. An estimated 90% of all cancer-related deaths are caused by cancer metastasis, making it a significant obstacle to effective cancer care^2^. Nearly 20% of CRC patients present with metastatic disease at initial diagnosis, and the liver is the most general metastatic site for CRC, accounting for almost 50% of all cases^3^. Metastasis of CRC leads to a poor prognosis^4^. Despite receiving standard treatments such as surgical removal, radiation therapy, and systemic chemotherapy, many patients with CRC liver metastasis still experience high rates of recurrence and less favorable clinical outcomes^2,5^. Hence, early diagnosis of liver metastases of CRC is crucial for improving patients’ prognosis and clinical outcomes^6^. Imaging examinations and focal biopsies are necessary for diagnosing CRC liver metastasis. However, the sensitivity of imaging techniques for CRC liver metastasis is still insufficient to accomplish the benefit of early diagnosis^7^. Incorporating biomarkers alongside imaging methods can significantly enhance the accuracy of detecting CRC liver metastasis^8^. Various biomarkers are utilized for detecting CRC liver metastasis, including but not limited to CEA, CA19-9, CA125, and others^9,10^. On the other hand, while serum markers such as CEA are helpful in diagnosing CRC, their limitations in terms of sensitivity and specificity decrease their reliability in identifying hepatic metastases particularly^6^. Thus, it is imperative to explore novel biomarkers to improve patients' diagnostic accuracy and clinical outcomes. Several types of biomarkers are used for cancer screening, including DNA, protein, and RNA biomarkers^11^. Transcriptional biomarkers are a promising class of biomarkers reflecting changes in the levels of RNA molecules produced from DNA in cells, including mRNAs, micro RNAs, long non-coding RNAs, and circular RNAs. They are non-invasive and highly sensitive, making them a valuable tool for the early detection and monitoring of various cancers^12^. Cancer initiation, development, and metastasis are influenced by complex processes and alterations at the transcriptome levels^13^.

Healthcare digitalization and the development of high-throughput technologies, such as microarray and next-generation sequencing (NGS), have enabled the collection of large amounts of transcriptome data in comprehensive databases such as The Gene Expression Omnibus (GEO) and The Cancer Genome Atlas (TCGA) database, which can be used to understand the underlying mechanisms of diseases which in turn developing precision medicine^14^. The analysis of transcriptome data can be complex and time-consuming, requiring expertise in bioinformatics and statistics. Traditional methods for analyzing transcriptome data involve manual curation and interpretation, which can be error-prone and may not be beneficial in handling the large amounts of data generated by modern sequencing technologies^15^. Artificial Intelligence (AI) algorithms such as machine learning (ML) can identify patterns and relationships in the data that would be difficult or impossible to detect using manual methods^16^. ML is a branch of AI that applies statistical methods and algorithms to achieve data parsing, categorization, and pattern recognition. This obtained information later enables computers to learn from data processing experiences and make more accurate predictions^17^. It is anticipated that ML will have a preeminent impact in the therapeutic context for detecting and treating cancer in the near future. It is worth mentioning that ML has been progressively utilized for the screening, diagnosis, and therapy of CRC over the course of the past five years^18^.

We used a machine learning-based feature selection technique to explore liver metastasis-related biomarkers in CRC. These methods are particularly beneficial for handling high-dimensional data and complex interactions between features and improving predictive models' performance which, results in identifying relevant features based on their predictive power. In the present study, features commonly selected by two feature selection algorithms were investigated through in-silico and experimental validation.

## Materials and methods

### Study design, data resources, and preprocessing

**Figure 1:**
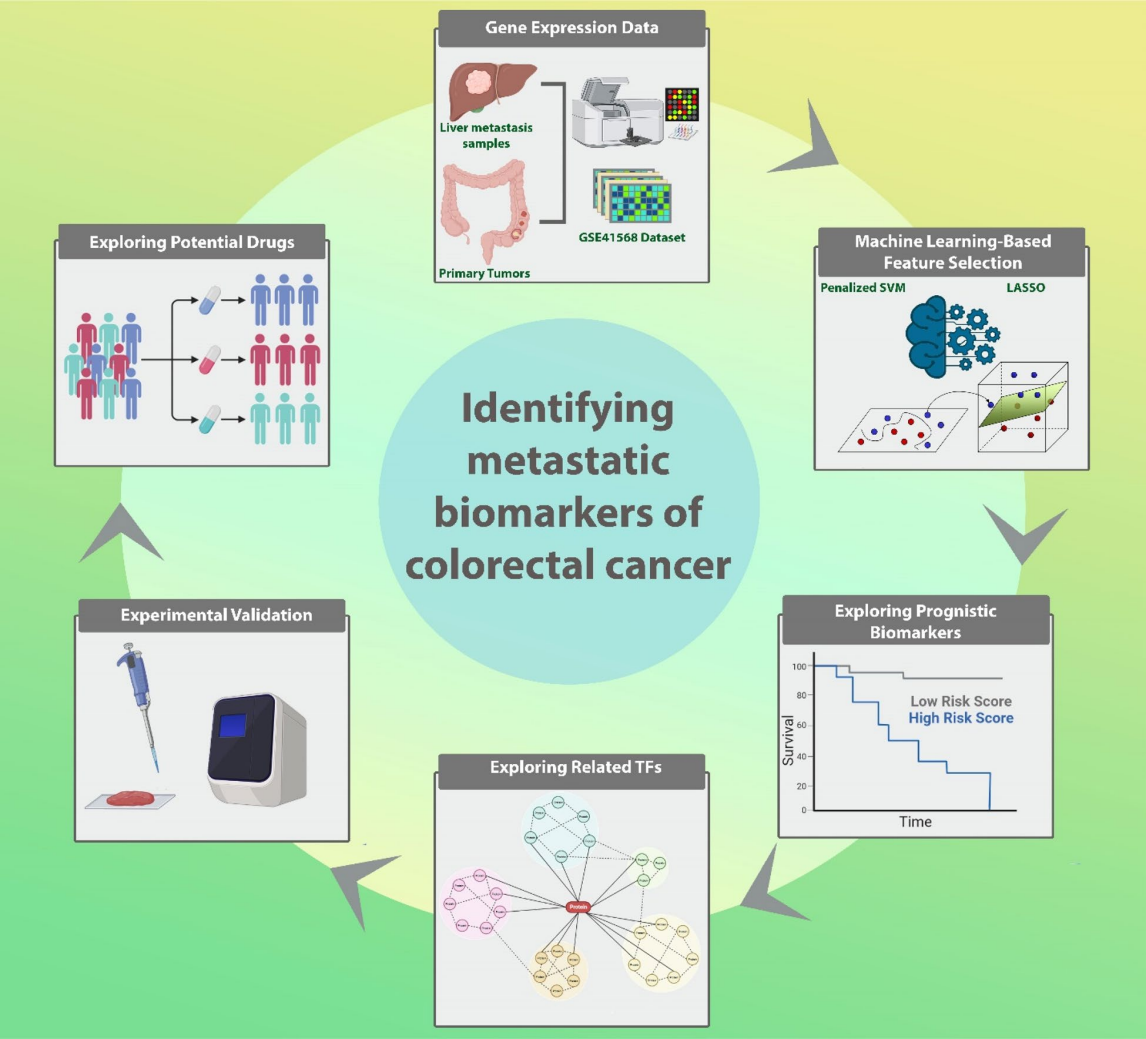
GSE41568 dataset was analyzed for identifying differentially expressed mRNAs (DEGs) in primary CRC samples and liver metastases. Next, using ML-based feature selection methods, most relevant DEGs were selected. Further analyses such as survival analysis and their potential targeting drugs and related TFs were investigated. Experimental validation was also carried out as the last step of our study.

A schematic representation of the research process is illustrated in Fig. 1. Primary search was conducted using the terms "colorectal neoplasms" and metastasis in the Gene Expression Omnibus database (GEO, http://www.ncbi.nlm.nih.gov/geo) to identify any relevant available datasets based on these criteria: (1) datasets contain primary CRC and metastatic liver tumor samples; (2) comprise more than 20 samples in each group; (3) more than 10,000 genes in each dataset. Based on these considerations, three microarray datasets, GSE41568, GSE41258, and GSE68468, were included in the present study (Table 1). GSE41568 dataset (GPL570, Affymetrix Human Genome U133 Plus 2.0 Array) containing 80 liver metastases and 39 primary tumors was selected as the main dataset. GSE41258 (GPL96, Affymetrix Human Genome U133A Array) with 186 primary tumors and 67 liver metastases and GSE68468 (GPL96, Affymetrix Human Genome U133A Array) with 186 primary tumors and 47 liver metastases were considered as validation datasets. In addition, we used the TCGA database (https://portal.gdc.cancer.gov/) to obtain RNA-seq data and clinical details of 644 CRC patients (89 metastatic (M1) and 555 non-metastatic (M0) samples) as well as another validation set in our study.

**Table 1:** Characteristics of the GEO datasets.

Accession number	Platform	# Samples (primary/metastatic)	DEGs
GSE41258	GPL97	253 (186/67)	85
GSE68468	GPL96	252 (185/67)	138
GSE41568	GPL570	133 (39/94)	496

On each platform, raw data was retrieved for these three datasets. All datasets were normalized if needed in R software(version 3.6.0; https://www.r-project.org/)^19^. Raw data was also evaluated to contain logarithmic fold change values, and if necessary, logarithm 2 of values was obtained. The probe IDs were converted into gene expression symbols on each annotation platform. Multiple probes related to a single gene were averaged to give the gene expression value. Also, probes with a vacancy were removed.

### Gene set enrichment analysis (GSEA)

GSEA is a popular method for identifying genes' biological significance by analyzing gene set expression patterns. This approach holds the potential to offer valuable insights into underlying biological processes that are associated with expressed genes. In this regard, we used GSEA software (version 4.1.0) to conduct this analysis to uncover the pathways most relevant to all expressed genes between primary tumors and liver metastases of CRC patients in the GSE41568 dataset by setting FDR criteria to < 0.05.

### Screening of differentially expressed genes (DEGs)

To screen DEGs between primary tumors and liver metastasis samples in the GSE41568 dataset, we carried out differential gene expression analysis using the limma package^20^ in R. |log2Fold Change |≥ 1 and False Discovery Rate threshold (FDR) < 0.05 were considered as cut-off criteria.

### Gene ontology (GO) and KEGG pathway enrichment analysis of DEGs

To investigate the biological properties of these DEGs, KEGG (Kyoto Encyclopedia of Genes and Genomes (http://www.Kegg.jp)) and GO enrichment analysis of selected DEGs was carried out using ClusterProfiler^21^, and GOplot^22^ packages in R. Statistical significance was assigned in terms of Benjamini < 0.05.

### Feature selection using machine learning algorithms

ML methods are tools to develop and evaluate classification and prediction algorithms. Data collection, model selection, training the model, and testing the model are the four steps that make up the foundation of machine learning^23^. Large numbers of input characteristics are challenging for ML methods to manage. Consequently, data preparation is a necessary task for supporting the use of machine learning in real-world settings. Feature selection is among the most used data preparation techniques for screening outcome-related variables from a large pool of variables^24^. Obtaining the appropriate features or subsets of elements from the literature to fulfill their classification goals has become an essential part of the ML procedure. In addition to the benefits of feature selection processes to search for a subset of important features, they are also employed to prevent overfitting and produce more efficient models^23^. We used two feature selection algorithms in the present study to pick cancer-related genes that accurately discriminate metastatic samples from non-metastatic ones, including 1). Random Forest (RF), 2). Penalized Support Vector Machine (P-SVM) with two penalties of Smoothly Clipped Absolute Deviation (SCAD) and Least Absolute Shrinkage and Selection Operator (LASSO).

### Random Forest

Random Forest, proposed by Breiman (2001), is a well-known technique that belongs to the ensemble algorithms used for classification and regression issues. This method forecasts an outcome by averaging the results of hundreds or more decision trees. RF is also employed as a variable selection strategy to identify informative variables^25^. The "randomForestSRC" R package was utilized in our investigation to determine the best features.

### Penalized support vector machine

Support vector machine (SVM) classification is one of the most popular and effective classification approaches^26^. However, a significant shortcoming of this method is that it cannot perform automated gene selection. To handle this problem, the " P-SVM" method was introduced, including two wrapper feature selection techniques for SVM classification utilizing the penalty function^27^.

Let us consider $\left\{\left({x}_{1},{y}_{1}\right),\dots ,\left({x}_{n},{y}_{n}\right)\right\},{x}_{i}\in {\mathfrak{R}}^{d},{y}_{i}\in \{-\mathrm{1,1}\}$ be the training data (x is the input data, and y is the binary outcome variable. The following linear boundary function separates the classes of the outcome variable in the linear SVM problem:

$$f(x)=\sum_{j=1}^{d} {w}_{j}{x}_{j}+b$$

The "wj" is the regression coefficient of the obtained hyperplane, and "b" stands for its intercept.

Then the assignment rule for the test dataset to each class is given as: ${y}_{\text{test }}=\mathrm{sign}\left[f\left({x}_{\text{test}}\right)\right]$

In the above problem, the finding of the optimal hyperplane is conducted by convex optimization. In the penalized version, maximizing the margins or optimization is achieved by the following penalized problem:

$$\underset{b,w}{min} \sum {\left[1-{y}_{i}f\left({x}_{i}\right)\right]}_{+}+{\mathrm{pen}}_{\lambda }(w)$$

Here the ${\mathrm{pen}}_{\lambda }(w)$ is the LASSO (least absolute shrinkage and selection operator) and SCAD (Smoothly Clipped Absolute Deviation).

### Penalized logistic regression

The logistic regression model considers a linear relationship between predictors (here, gene profiles) with a binary (dichotomous) outcome (here, having colorectal cancer liver metastasis or being a healthy control)^28^. So, the outcome, which variable takes y = 1 or y = 0, is considered to have a Bernoulli distribution with $P(y=1)=\pi =\frac{\mathrm{exp}\left({\beta }^{T}X\right)}{1+\mathrm{exp}\left({\beta }^{T}X\right)}$. Here, $\beta$ is the vector of regression coefficients, including an intercept term, and X is the data matrix of the gene expression profile of the patients and healthy control. Therefore, considering the logit transform, we have the following regression form:

$$\mathrm{log}it(\pi )=\mathrm{log}\left(\frac{\pi }{1-\pi }\right)={\beta }^{T}X$$

Then, the parameter estimation is conducted by the maximum likelihood estimation by considering the following log-likelihood function:

$$\mathrm{log}(L(y;\beta ))=\sum_{i=1}^{n} \left\{{y}_{i}\mathrm{log}\left({\pi }_{i}\right)+\left(1-{y}_{i}\right)\mathrm{log}\left(1-{\pi }_{i}\right)\right)$$

Like the P-SVM, to handle the high dimension problem (having a greater number of predictors than the sample size), the penalized likelihood is used for variable selection. The penalty terms can have different forms. Here, we considered Lasso and SCAD (Smoothly Clipped Absolute Deviation).

### LASSO and SCAD penalties

The LASSO approach imposes a constraint on the total of the absolute values of the model parameters; the sum must be smaller than a predetermined value^29^: (in the equation below, λ ≥ 0 is the tuning parameter).

In terms of variable selection, LASSO and SCAD have similar performances^30^. SCAD penalty, suggested by Fan and Li in 2001^31^, is used to reduce the bias while estimating large regression coefficients^32^:

$${p}_{\lambda }\left({\beta }_{j};a\right)=\left\{\begin{array}{ll}\lambda \left|{\beta }_{j}\right|& \left|{\beta }_{j}\right|\le \lambda \\ -\left(\frac{{\beta }_{j}^{2}-2a\lambda {\beta }_{j}\mid +{\lambda }^{2}}{2(a-1)}\right)& \lambda <\left|{\beta }_{j}\right|\le a\lambda \\ \frac{(a+1){\lambda }^{2}}{2}& \left|{\beta }_{j}\right|>a\lambda \end{array}\right.$$

The tuning parameters in this equation are λ ≥ 0 and *a* > 2. In this study, we set the tuning parameter α's value at 3.7. However, the lambda parameter was tuned by the cross-validation method.

### Evaluating the performance of feature selection techniques

In order to assess the efficiency of genes selected by each method in differentiating primary samples from metastatic tumors, we applied the Artificial Neural Network (ANN) method using the Multilayer Perceptron procedure in SPSS 24.0. Algorithms with an area under the ROC curve (AUC) > 0.9 were opted, and commonly selected features by these algorithms were considered as the main DEGs for further analysis.

### Establishment of the SVM model

SVM is a supervised ML algorithm that is mainly used for data categorization^33^. This algorithm distinguishes sample type by estimating the degree of a sample that belongs to a specific class^34^. As a part of this study, We constructed an SVM classifier based on selected features for the GSE41568 training set using the "e1071" package in R^35^. The SVM classifier's efficacy was assessed on the training and three independent validation sets (GSE68468, GSE41258, and TCGA COAD-READ).

### Survival analysis to identify genes with prognostic value

Disease-free survival (DFS) and overall survival (OS) analyses were carried out to identify genes with prognostic significance. In this regard, for OS analysis, 644 TCGA COAD-READ samples were categorized into high-expression and low-expression groups based on the optimal cut-off points determined by the "survminer" and "maxstat" R packages. Using the "survival" package and P value < 0.05, we conducted a Kaplan–Meier survival analysis with a log-rank test to determine which genes are associated with overall survival.

### Transcription factor-DEGs network construction

Toward identifying the transcription factors (TFs) of the key genes, we utilized the NetworkAnalyst online tool^36^. NetworkAnalyst is a web-based application for comprehensive gene expression profiling and meta-analysis using network-based visual analytics^36^. To construct the TF-gene network, the final DEGs were submitted to NetworkAnalyst to collect information on TF-gene and microRNA-gene interactions, and the resultant list of datasets was exported to Cytoscape software (version 3.7.1) for additional analysis.

### Drug–DEGs interaction network

The Drug Gene Interaction Database (DGIdb) (https://www.dgidb.org/) was utilized to find the potential drugs that target the final genes. This database is linked to 22 different databases. To find drug–DEG interactions in the current investigation, only empirically verified interactions were examined.

### Sample collection

40 CRC samples, including 16 stage IV CRC samples and 24 CRC samples from other stages, were obtained from Iranian patients who underwent surgery in Mortaz Hospital in Yazd, Iran. All tumor samples were preserved at – 80 °C until the RNA extraction process. (Table 2). Also, five liver metastasis paraffin-embedded samples were received from the archive of the Cancer Institute of Imam Khomeini Hospital. The Study procedure was authorized by The Hamadan University of Medical Science Ethics Committee (ethical code: IR.UMSHA.REC.1400.530). Additionally, informed consent was acquired from all participating patients in this study. The procedures were carried out in compliance with the Helsinki Declaration's laws and recommendations.

**Table 2:** Demographic information of patients.

	n	%
Age		
≤ 60	14	35
> 60	26	65
Gender		
Male	19	48
Female	21	52
Stage		
I	4	10
II	12	30
III	8	20
IV	16	40
Grade		
I	18	45
II	15	37
III	7	17

### Experimental validation: real-time PCR assay

We extracted total cellular RNA from both fresh frozen and paraffin-embedded tissues using the RNX kit (RNX, Cina Gene Company, Iran). Then Yekta Tajhiz cDNA Synthesis Kit (Yekta Tajhiz Azama, Iran) was used to convert the extracted RNA into cDNA. The quantitative reverse transcription PCR (RT-qPCR) was carried out on each sample in duplicate using SYBR Green Master Mix Kit (©Ampliqon, Herlev, Denmark) in a LightCycler 96 Real-Time PCR detection system (Roche, United States) in accordance with the manufacturer’s guidelines. The primer sequence of four evaluated genes in this study is presented in Table 3. Among 11 feature genes, we selected four with the highest AUC in the SVM model (Supplementary Figure 1) and prognostic value. In this analysis, GAPDH was used as the reference gene. Finally, we calculated the relative expression levels of the genes using the 2^−△△Ct^ method^37^. The gene expression levels were assessed and compared among three distinct groups: Stage IVCRC samples, CRC samples from stages I to III, and liver metastasis samples.

**Table 3:** The sequence of the primers and characteristics of studied genes.

Gene name	Gene ID	Forward primer	Reverse primer	Product size (bp)
MMP3	4314	5′GAACAATGGACAAAGGATACAAC3′	5′TTGGCTGAGTGAAAGAGACC3′	92
TNFSF11	8600	5′TCACAGCACATCAGAGCAGAG3′	5′GACAGACTCACTTTATGGGAACC3′	146
WNT11	7481	5′TCCCAAGCCAATAAACTGATG3′	5′CTTACACTTCATTTCCAGAGAGG3′	84
WNT5A	7474	5′GCAATGTCTTCCAAGTTCTTCC3′	5′CATACCTAGCGACCACCAAG3′	96
GAPDH	2597	5′AAGGCTGTGGGCAAGGTCATC3′	5′GCGTCAAAGGTGGAGGAGTGG3′	248

### Statistical analysis

The data were analyzed utilizing the R programming language, SPSS 24.0, and the GraphPad Prism 9.0 software. The differences between expression values of the three sample groups were evaluated through a one-way ANOVA test. Statistical significance was determined using the thresholds of *P value < 0.05, **P value < 0.01, and ***P value < 0.001 for all statistical tests.

### Ethical approval

Ethical issues (Including plagiarism, informed consent, misconduct, data fabrication and/or falsification, double publication and/or submission, redundancy, etc.) have been completely observed by the authors.

The ethical protocol of this study was approved by the Ethics Committee of Hamadan University of Medical Sciences. (Ethical code: IR.UMSHA.REC.1400.530.) and written informed consent was obtained from all patients to participate in the study.

## Results

### Identification of DEGs related to CRC liver metastasis in the datasets

**Figure 2:**
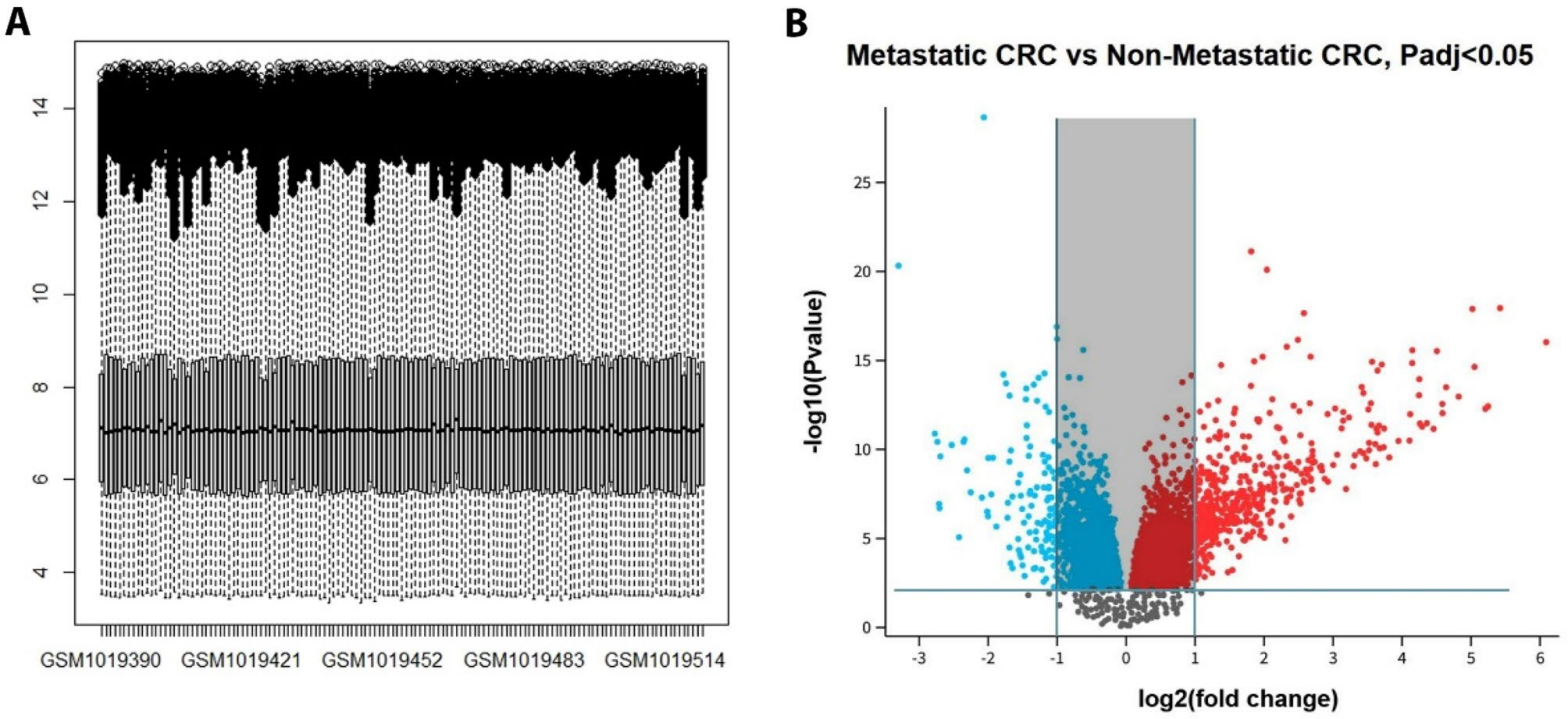
Identification of DEGs between metastatic and primary tumors. (**A**) The box plot of gene expression levels in GSE41568. (**B**) The volcano plot of DEGs.

**Figure 3:**
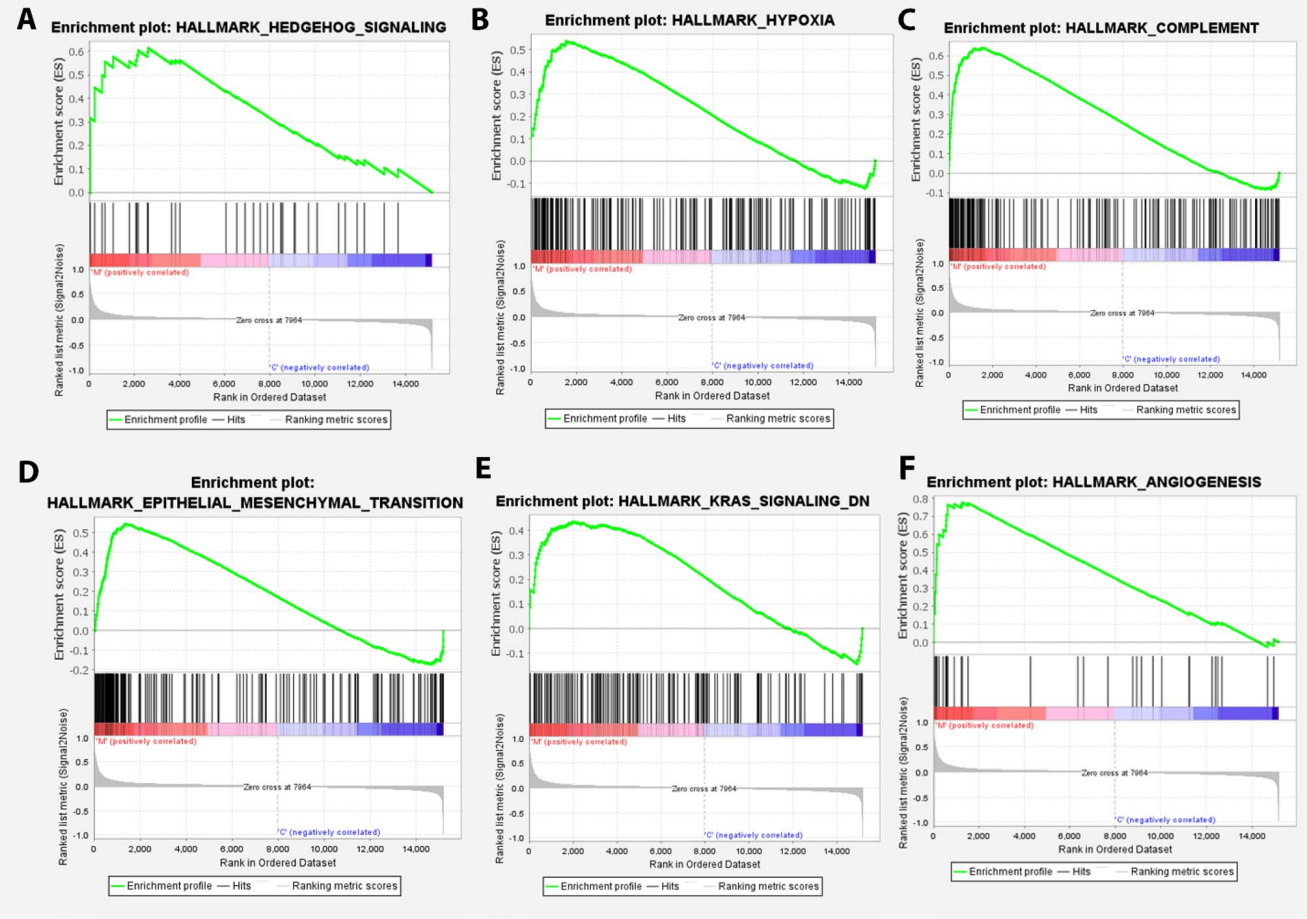
Gene set enrichment analysis of all expressed genes between primary and metastatic CRC patients in the GSE41568. This analysis demonstrated that these genes are enriched in (**A**) Hedgehog signaling, (**B**) Hypoxia, (**C**) Complement, (**D**) Epithelial-Mesenchymal Transition, (**E**) KRAS signaling Down, and (**F**) Angiogenesis pathways with nominal P value < 0.05.

Details of selected databases are summarized in Table 1. The gene expression levels of selected samples are shown in Fig. 2. Samples in the GSE41568 dataset were divided into two groups: primary tumor and metastatic tumor, and gene expression analysis was carried out (cut-off criteria: |logFC|≥ 1 and FDR < 0.05). This analysis identified 496 DEGs containing 393 upregulated and 103 downregulated genes, comparing metastatic and non-metastatic samples. To investigate the biological functions of all expressed genes between primary and metastatic CRC patients in the GSE41568 dataset, the GSEA method was employed using the ‘hallmarks’ gene set. As shown in Fig. 3, these genes were enriched in pathways including “Hedgehog signaling”, “Hypoxia”, “ Complement”, “ Epithelial-Mesenchymal Transition”, “ KRAS signaling Down”, and “ Angiogenesis” pathways (nominal P value < 0.05).

### GO, KEGG pathway analysis

**Figure 4:**
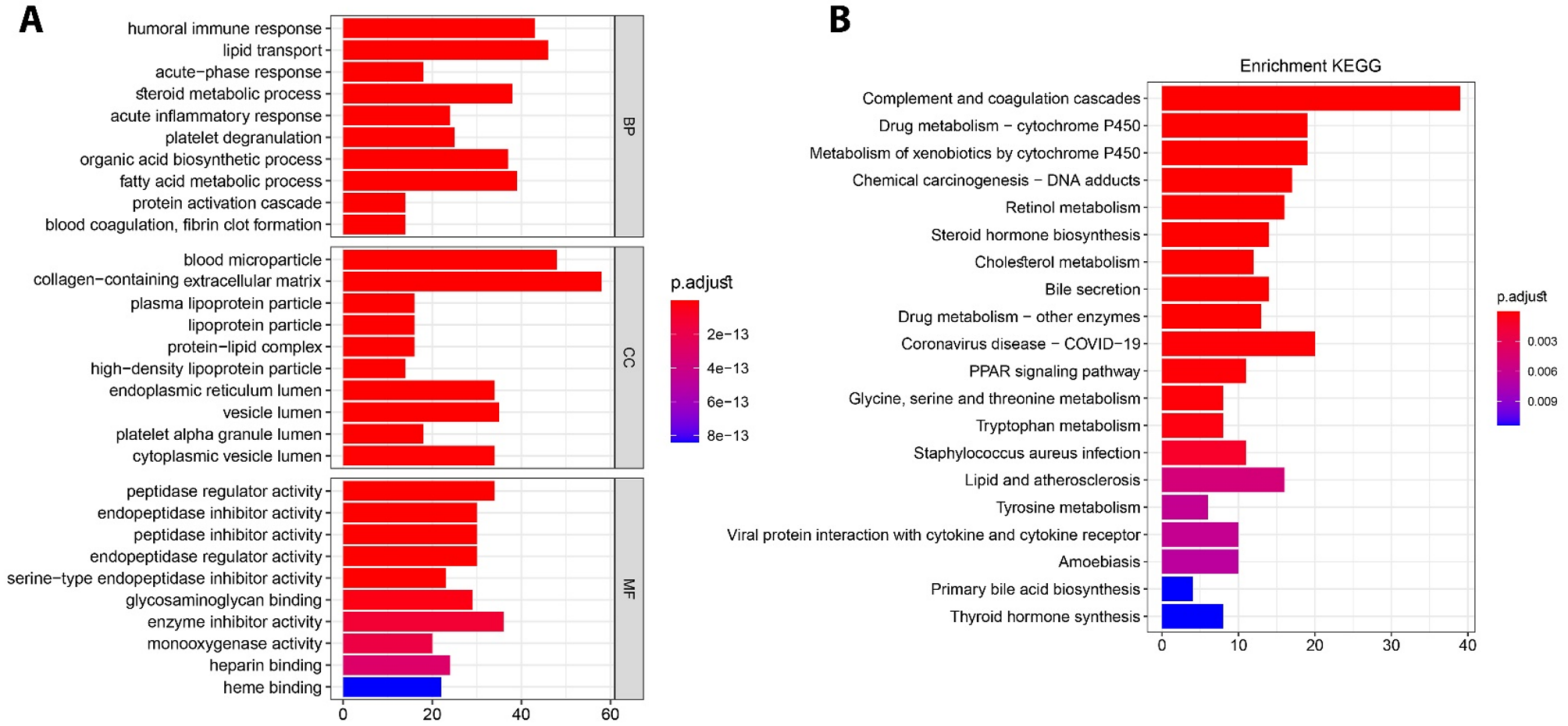
GO and KEGG pathway enrichment analyses of DEGs using ClusterProfiler. Results of (**A**) biological process, cellular component and molecular function as well as (**B**) KEGG pathway enrichment analyses.

GO categorizes genes according to their molecular function (MF), biological process (BP), and cellular component (CC)^38^. The DEGs were most strongly related to the BP of “humoral immune response” (GO:0006959), CC of “blood microparticle” (GO:0072562), and MF of “peptidase regulatory activity” (GO:0052547), according to the analysis of the GO terms. Remarkably, most of the top enriched gene ontology BP, CC, and MF terms were related to a lipid metabolic process, including the “fatty acid metabolic process” and “peptidase activity”. Moreover, the screened DEGs were considerably enriched in the “complement and coagulation cascade pathway” (Fig. 4).

### Selecting the features

Machine learning algorithms of RF, P-SVM, and logistic regressions with LASSO and SCAD penalties were employed for selecting features associated with metastasis among 496 screened DEGs. The LASSO method applied by the “glmnet”, P-SVM method by the “penalizedSVM” package, and SCAD by “grpreg” package in R selected 20, 32, and 6 features, respectively, as the most relevant features. Also, 43 features were selected by random forest algorithm. Genes selected by each algorithm are listed in supplementary table 1.

### Using ANN to compare variable selection methods

**Figure 5:**
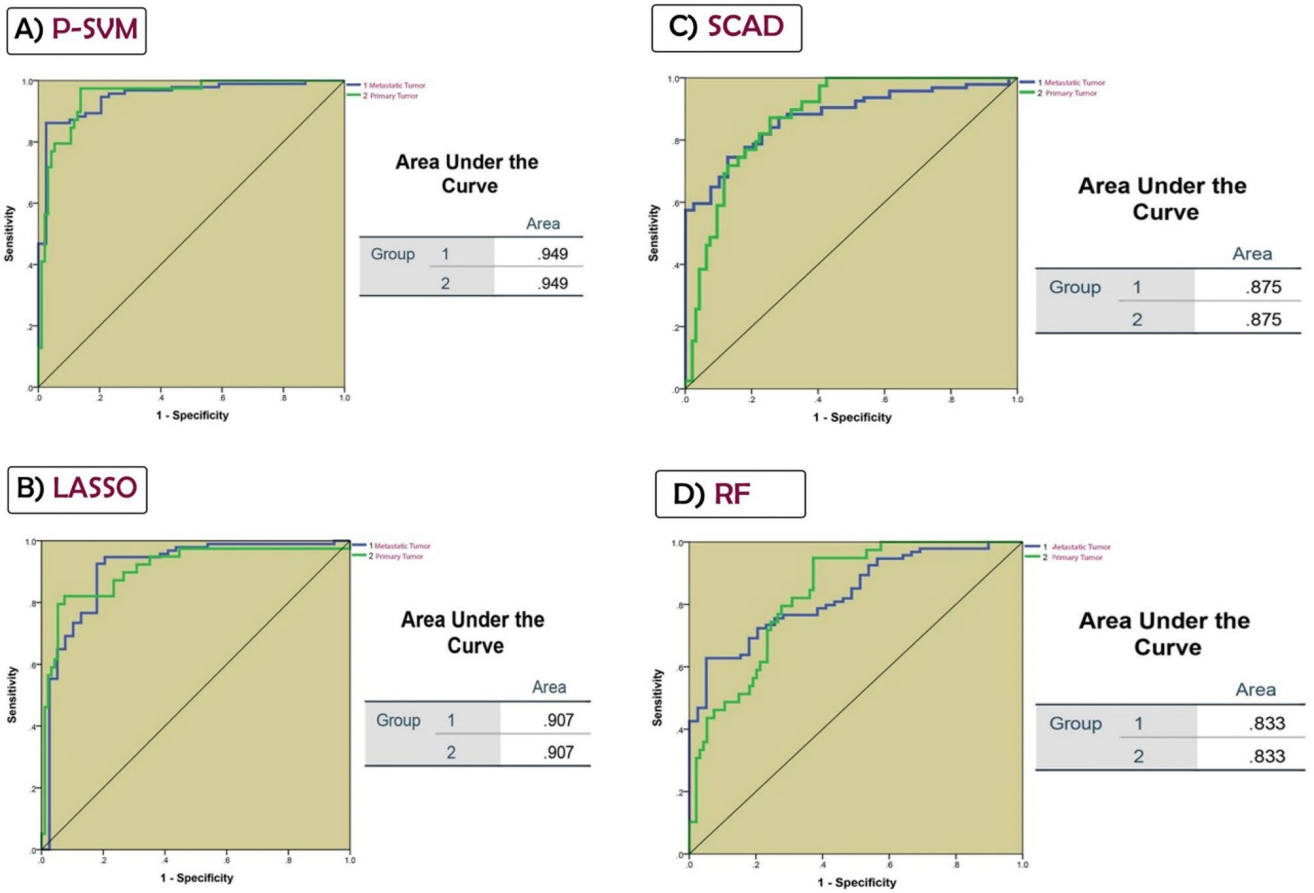
ROC curves of ANN models constructed based on features selected by (**A**) P-SVM, (**B**) LASSO, (**C**) SCAD and, (**D**) RF. AUC of each algorithm is presented in this figure. Based on this results P-SVM and LASSO were selected as main feature selection methods in our study.

A multi-layer perceptron artificial neural network was utilized to compare the accuracy of feature selection methods. In this regard, ANN was trained with 70% of the data based on features selected by each algorithm, a hold-out validation technique was applied in SPSS, and the performance of the algorithms was evaluated with respect to the area under the receiver operating characteristic (ROC) curve. The AUC results revealed that P-SVM and LASSO are the most accurate models, with an AUC of 0.94 and 0.9, respectively. Roc curves of different algorithms are presented in Fig. 5. Features picked by both LASSO and P-SVM algorithms were considered as the main features for this study which were *MMP3, TNFSF11, WNT5A, EPHA3, WNT11, CXCR4, MAP2, MAB21L2, FOXC1, TMEM158, PDE4D*. These 11 genes may have the potential to be considered as a diagnostic panel for colorectal cancer metastasis.

### Development and verification of predictive SVM model based on feature genes

**Figure 6:**
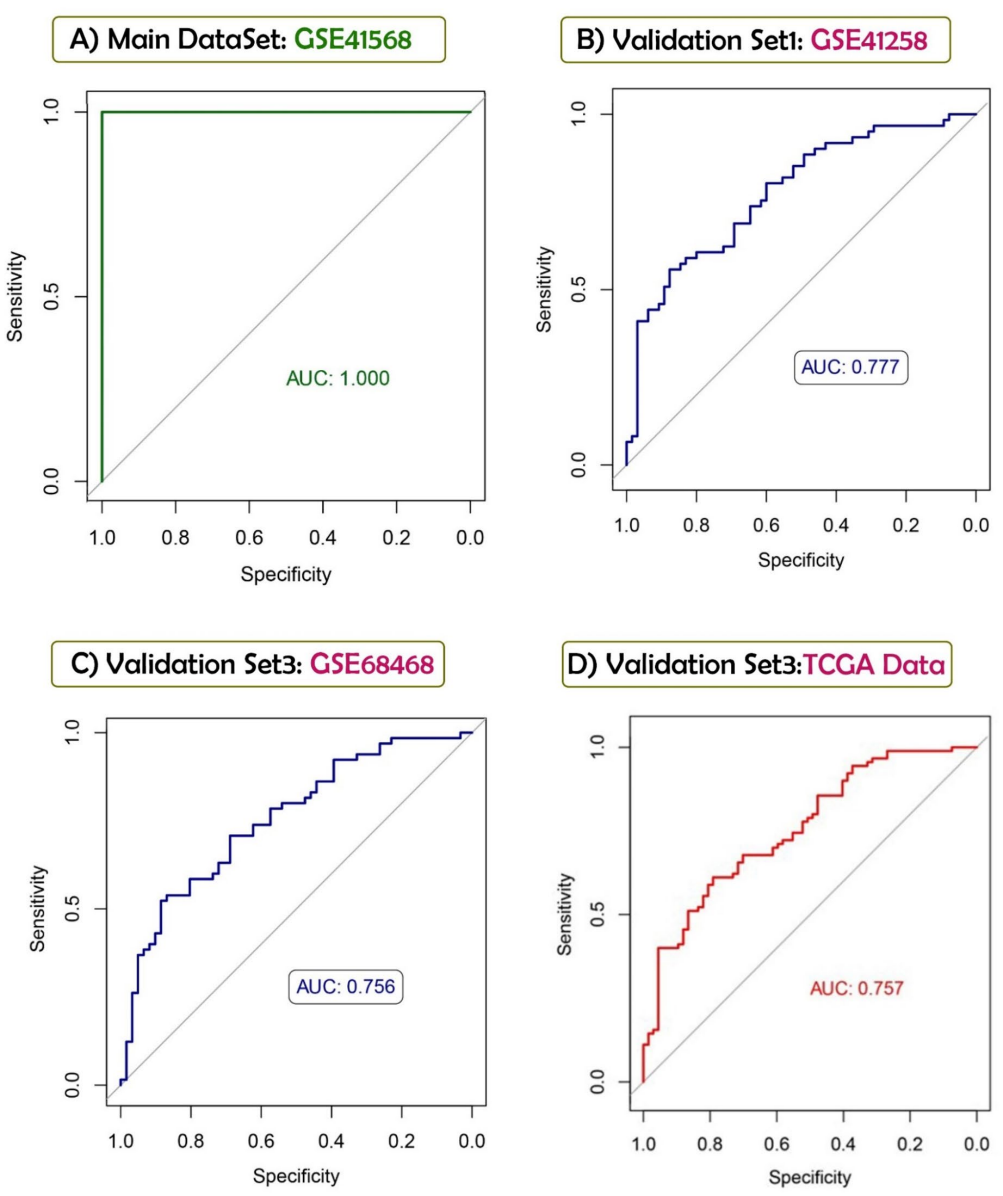
Predictive SVM models based on 11 DEGs selected by LASSO and P-SVM using (**A**) GSE41568 as training set and (**B**) GSE41258, (**C**) GSE68468 and, (**D**) TCGA COAD-READ (M0 vs. M1) as validation sets.

The 11 mentioned (previous paragraph) genes from the GSE41568 training dataset were used to construct an SVM predictive classification model. The C-Classification SVM method was applied with Radial Based Function (RBF) kernel and tenfold cross-validation. The AUC in the GSE41568 was 1, standing for sensitivity and specificity of 100% (Fig. 6). We also used three external datasets (GSE68468, GSE41258, and TCGA COAD-READ) to evaluate the model. In the GSE68468 and the GSE41258 validation sets, the AUC was 0.75 and 0.77, respectively. TCGA COAD-READ dataset was used as the other validation set. We divided the samples into two groups (M0 and M1) based on the TNM staging characteristics of each sample. The AUC for this validation dataset was 0.75.

### Identification of genes with prognostic value

**Figure 7:**
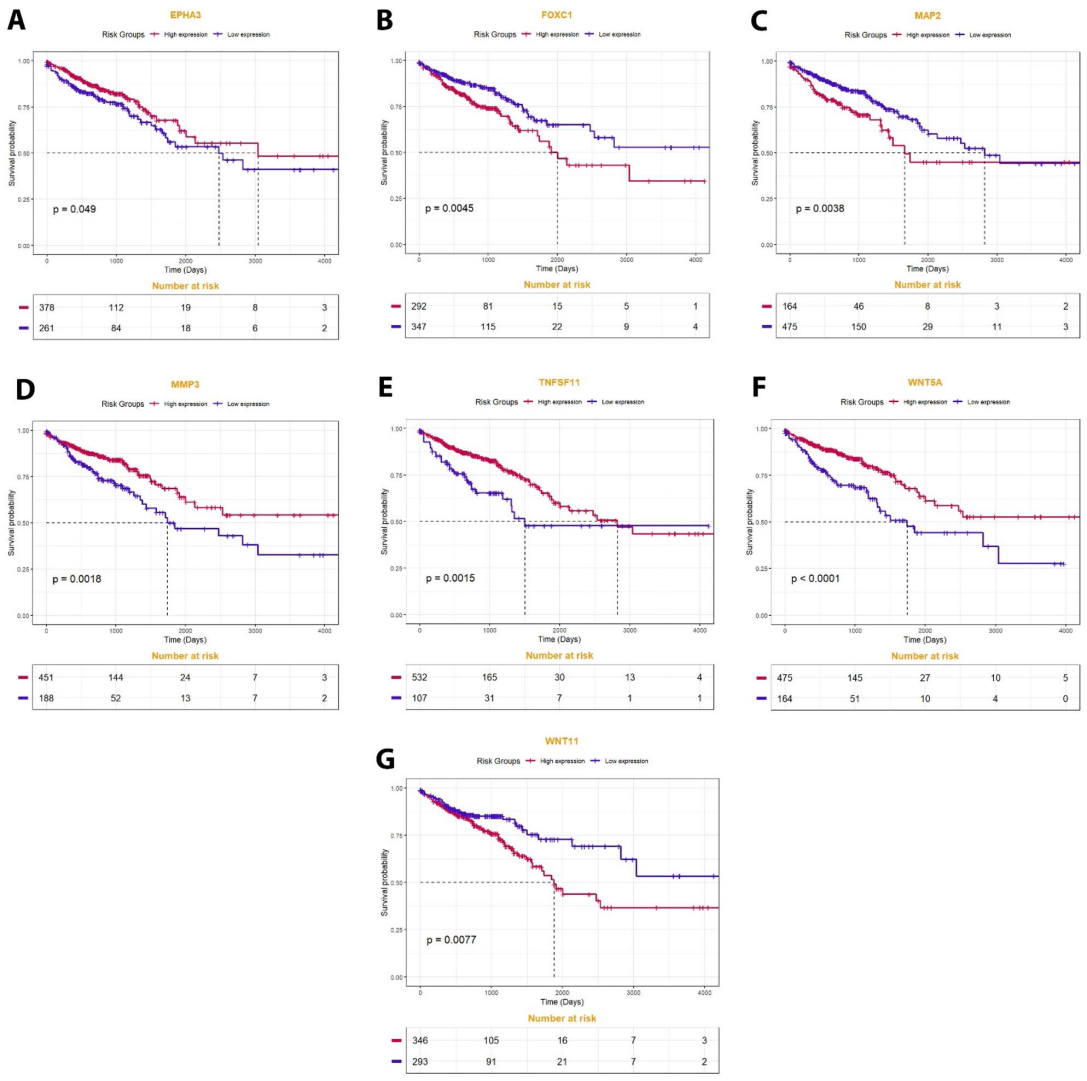
The association between expression of (**A**) EPHA3 (P value = 0.049), (**B**) FOXC1 (P value = 0.0045), (**C**) MAP2 (P value = 0.0038), (**D**) MMP3 (P value = 0.0018), (**E**) TNFSF11 (P value = 0.0015), (**F**) WNT5a (P value < 0.0001), and (**G**) WNT11 (P value = 0.0077) with overall survival of all patients in the TCGA COAD-READ dataset. The red line indicates high expression groups and the blue line represents the low expression group.

Survival analysis was conducted by the optimal cut-off value. The results showed that seven of eleven feature genes were significantly related to the poor prognosis of CRC patients. In this case, *WNT5a* (P value < 0.0001), *TNFSF11* (P value = 0.0015), *MMP3* (P value = 0.0018), and *MAP2* (P value = 0.0038) were the most significant genes predicting poor OS in CRC patients. The findings show that OS is lower in patients with low expression of *MMP3*, *WNT5a,* and *TNFSF11*. Also, patients with high expression of *WNT11* had lower OS. The association between the expression of these genes with OS of CRC patients is presented in Fig. 7.

### Transcription factors modulating feature genes

**Figure 8:**
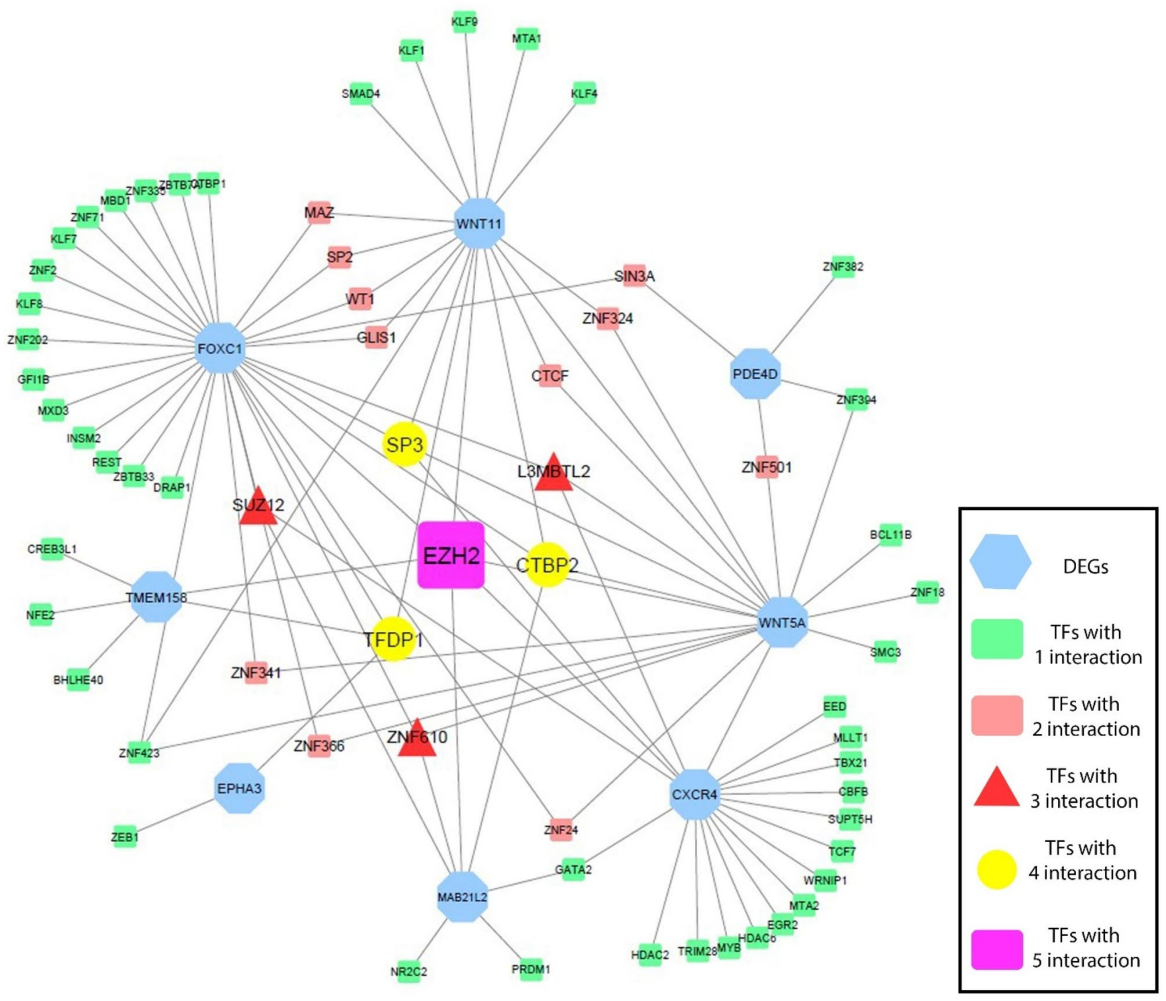
The TF–DEGs interaction network. According to this network,65 TFs were found to be interacting with seven of selected genes. Among them EZH2 interacts with five genes and could be considered as the most important TF in this network. TFs are shown in different colors standing for their importance and interaction (this figure was drawn in the Cytoscape v.3.8.2 software).

To uncover other underlying TFs regulating the selected genes, the TF–DEGs network was constructed using the networkanalyst tool and ENCODE database. According to this database, a total of 65 TFs were found to be related to the feature genes. The constructed network was visualized by Cytoscape and is depicted in Fig. 8. Seven genes had known interacting transcription factors. The resultant network shows that EZH2, the most interacting transcription factor in this network, regulates five feature genes.

### Drug-DEGs network

**Figure 9:**
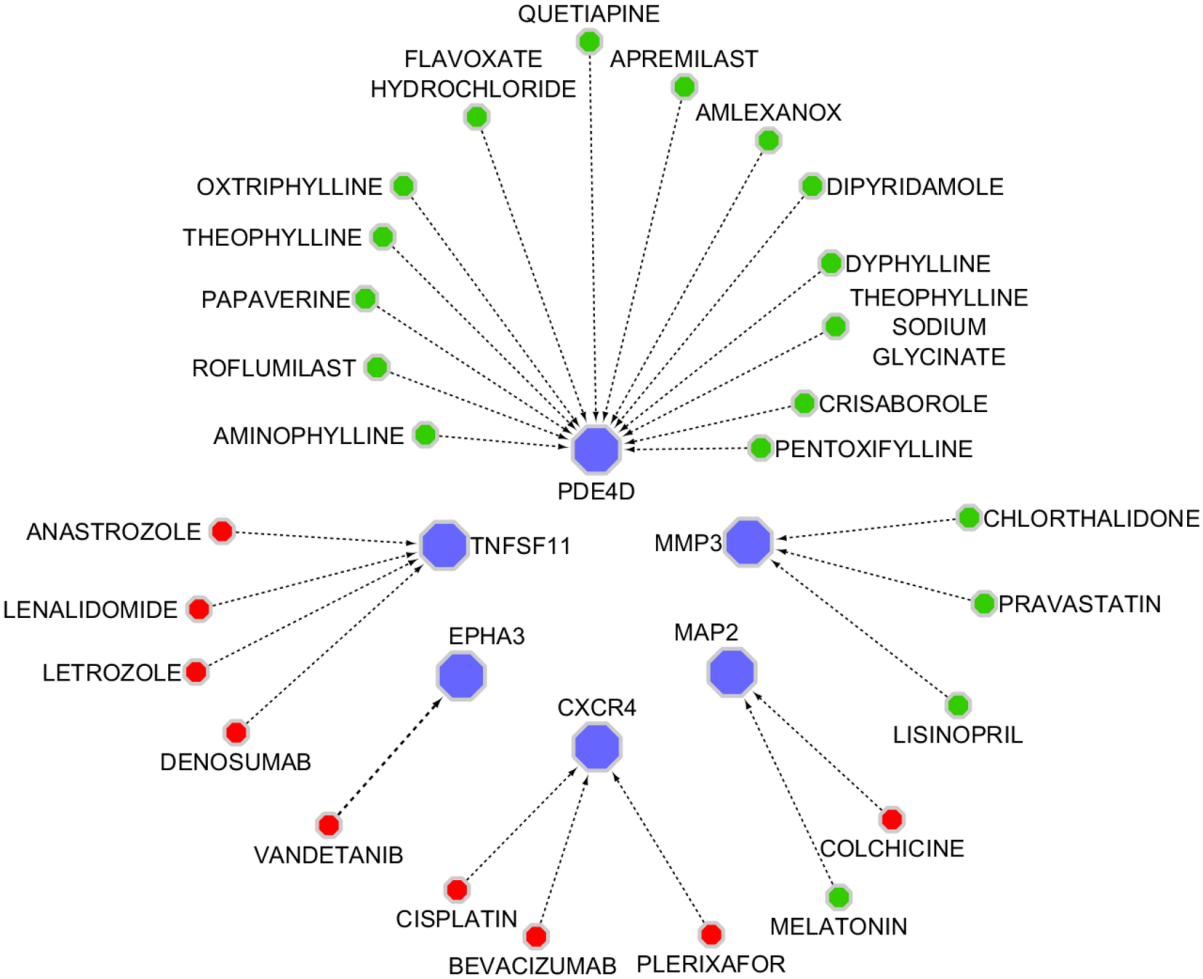
Illustration of the drug–gene interaction network. Totally, 27 candidate drugs were identified as modulators of the selected genes using DGIdb database. The red and green circles represent drugs and blue hexagon shapes represent genes. Red circle represents antineoplastic drugs. (this figure was drawn in the Cytoscape43 v.3.8.2 software).

The list of selected genes was imported into the DGIdb database to investigate any FDA-approved drugs related to these genes. Among feature genes *MMP3, EPHA3, MAP2, TNFSF11, CXCR4* and *PDE4D* had approved targeting drugs. A total of 30 drugs were found, nine of which were antineoplastic, including Lenalidomide, Anastrozole, Letrozole, Colchicine, Plerixafor, Bevacizumab, Cisplatin, and Vandetanib. The drug-gene network was illustrated using Cytoscape and is represented in Fig. 9.

### Experimental validation using qRT-PCR:

**Figure 10:**
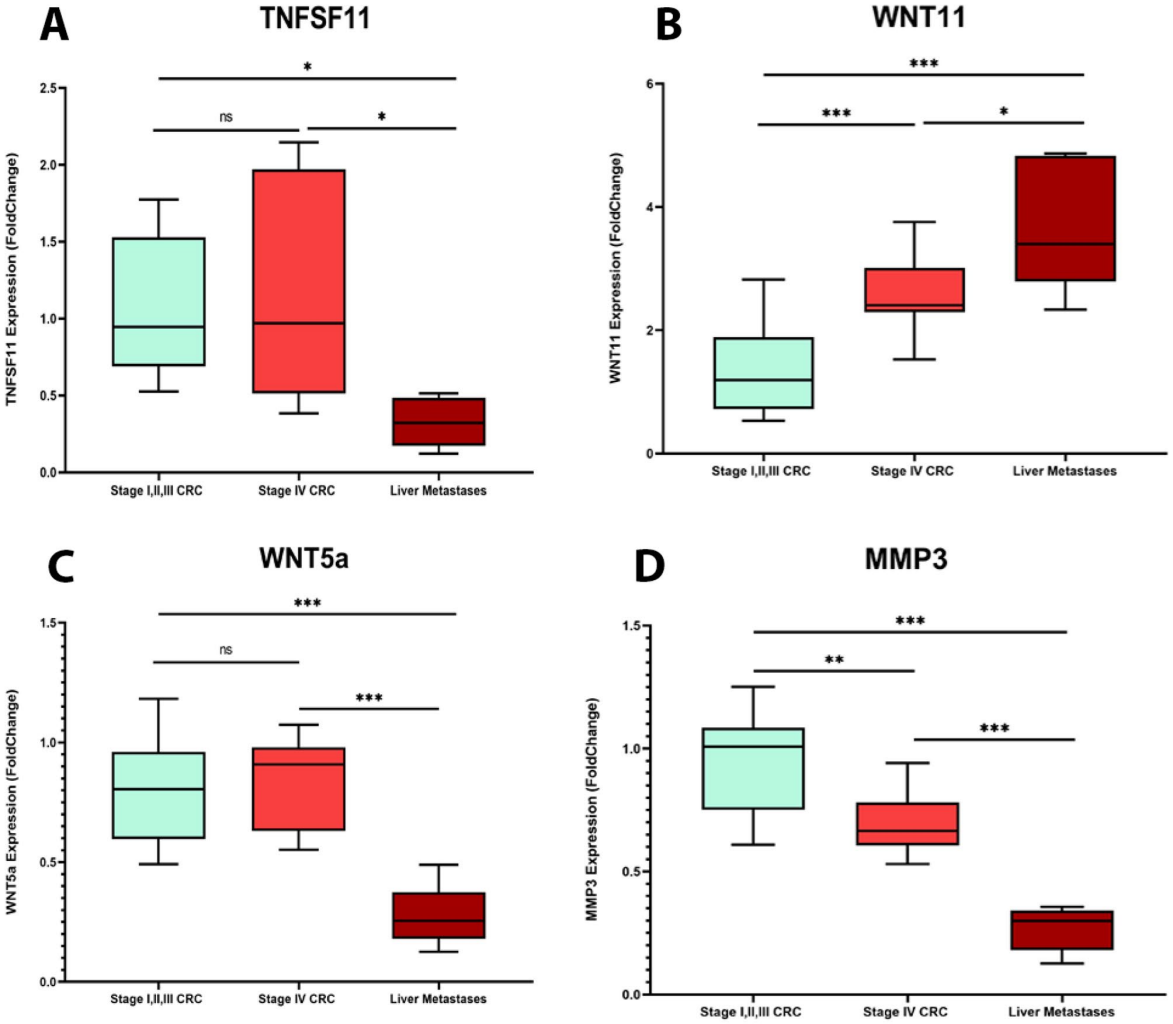
RT-qPCR analysis of gene expression of MMP3, TNFSF11, WNT5a and, WNT11 in stage IV (n = 16), stages I, II, III CRC samples (n = 26) and liver metastasis (n = 5) samples. (**A**) *TNFSF11*, (**B**) *WNT11*, (**C**) *WNT5a*, (**D**) *MMP3*. All data are presented in mean ± SD.

In the final section of our study, we investigated the gene expression levels of *MMP3*, *WNT5a*, *WNT11,* and *TNFSF11* genes. Analysis between three research groups revealed that expression of the *MMP3* gene was significantly lower in the liver metastasis group compared to other groups. The expression of this gene was also lower in the stage IV CRC group compared to CRC samples from other stages. *WNT11* was the other gene that showed a significant expression alternation in different groups. The results showed that this gene overexpressed significantly in liver metastases compared to stage 4 and stages 1,2,3 samples. This gene was also expressed in higher levels in stage 4 CRC samples compared to stages 1,2,3 CRC group. The gene expression levels of *WNT5a* and *TNFSF11* were also significantly lower in liver metastases compared to stage 4 and stages 1,2,3 CRC groups but showed no significant expression alternations between stage 4 and stages1,2,3 CRC samples (Fig. 10).

## Discussion

Ninety percent of cancer-related fatalities are due to metastatic spread^39^. Current cancer therapies are ineffectual for metastatic cancer because standard imaging tools cannot detect the disease in its early stages. In addition, the capacity to forecast cancer's ability to metastasize in advance will help to improve patient prognosis^40^. Therefore, it becomes crucial to investigate possible biomarkers with prognostic significance for metastatic CRC.

In the present study, we identified 11 distant metastasis-related genes using machine learning algorithms, seven of which substantially correlated with the survival of CRC patients. These genes were also utilized to construct an SVM predictive classification model with AUC = 1. In summary, 496 DEGs were screened by comparing the gene expression profiles of primary tumors with CRC liver metastases. KEGG enrichment analysis on screened DEGs revealed that “complement and coagulation cascades” is the most significant enriched pathway related to the primary DEGs. Recent studies have uncovered mounting evidence that complement and coagulation cascades are involved in angiogenesis, tumor cell proliferation, immune response suppression, and metastasis^41,42^.

Subsequently, we used two machine learning algorithms, P-SVM and LASSO, to select the features. Features picked by both algorithms were considered for further analysis. The results demonstrated that *MMP3*, *WNT11*, *WNT5a*, and *TNFSF11* might have essential roles in CRC metastasis. Among them, *WNT11* and *WNT5a*, WNT family members, regulate cell fate, proliferation, migration, and cell death in various ways and are engaged in the process of carcinogenesis and embryogenesis. Several studies have highlighted the significance of these genes in CRC development and metastasis. For instance, Fujii et al. found that *WNT5a* upregulation promotes the EMT in HT29 cells^43^. In another study, it was shown that *WNT5a* suppression by miR-21b initiates the metastatic process in CRC cells^44^. It's interesting to note that Ki et al.^45^ demonstrated that the *WNT5a* expression level is higher in primary tumors than in normal colon samples, while it has significantly lower expression in liver metastasis tumors. *WNT11* is another member of the WNT family. Noncanonical *WNT11* signaling promotes proliferation and morphological alternations in the intestinal epithelial cells. Several studies have revealed that the *WNT11* expression level elevates in CRC and increases the 5-year mortality rates^46–49^. This gene is positively related to cell migration and invasion in CRC cells, increasing the likelihood of metastasis. Gorroño-Etxebarria et al. proved that *WNT11* is highly expressed in CRC liver metastasis samples through immunohistochemical staining. Besides, some studies have demonstrated the significance of *WNT11* in other cancers. For example, Arisen et al. indicated that upregulated *WNT11* promotes EMT in aggressive prostate cancer cells^50^.

*TNFRSF11 (RANK)* was first found to have a role in bone dissolution and lymph node formation, majorly via the RANK/RANKL/OPG pathway^51^. Current studies have proved the critical role of RANK/RANKL/OPG in cell migration and invasion. Additionally, it has been observed that *TNFRSF11*(*RANK*) is engaged in the development of several types of cancers, including lung cancer^52^, prostate cancer^53^, renal cancer^54^, breast cancer^55^, and melanoma^56^. On the other hand, several investigations have shown that the RANKL/RANK system promotes both primary carcinogenesis and metastasis through osteoclast-independent mechanisms^57^. Furthermore, Ahern et al. found that *TNFSF11* knockdown enhances the anti-metastatic effect of antibodies targeting PD1/PD-L1 and suppresses the growth of the subcutaneous tumors of colon cancer animal models^58^.

*MMP3*, also known as *stromelysin-1*, is a member of matrix metalloproteinase (MMP). *MMP3* participates in several cellular biological processes, including cell differentiation and inflammation. This gene contributes to the onset and progression of several disorders. *MMP3* is capable of degrading ECM, which facilitates tumor invasion and metastasis. Different studies have revealed *MMP3* expression alternation and its role in metastasis in various cancers, such as osteosarcoma^59^ and ovarian cancer^60^. Moreover, *MMP3* expression was also discovered to be substantially elevated in malignant colorectal tumors compared with normal tissue^61^.

Interestingly, very few studies have shown that *MMP3* is downregulated in metastatic lesions compared with primary tumors of different cancers. Maiti et al. revealed that *MMP3* downregulates significantly in metastatic sites vs. primary breast tumors using qPCR (P value = 0.0001). In addition, they discovered a correlation between MMP3 and the prognosis of breast cancer patients^62^. Another study on this issue was conducted by wang et al. to figure out essential DEGs in CRC metastasis using NGS profiling on primary colorectal tumor samples from CRC patients with and without liver metastases and validated their findings by qPCR and immunostaining. The results of this research indicated that *MMP3* significantly downregulates in samples with liver metastasis^63^. The current study found clear support for these findings. We observed that the *MMP3* expression level was significantly lower in high-stage tumor samples compared with low-stage samples. This is in contrary to the findings claiming that the expression level of this gene is not associated with the tumor stages^64^.

The other part of our study was identifying vital transcription factors involved in CRC metastasis. We found that EZH2 modulates five interacting DEGs and could be considered an essential TF in this process. EZH2 is an inhibitory transcription factor that plays a role in the histone methylation process^65^. This protein takes part in the formation of heterochromatin structure, which causes gene silencing^66^. Various studies have been conducted to indicate EZH2 involvement in different cancers' progression and metastasis. In a study by Zheng et al., they represented that this transcription factor plays a direct role in breast cancer bone metastasis through the TGF-B pathway. They observed that *EZH2* knockout in mice prevented bone metastasis^67^. Also, Chen et al. reported that EZH2 is responsible for poor prognosis in CRC. They proved that EZH2 is upregulated in colorectal tumor tissues by qRT-PCR and western blot analysis. Additionally, they observed that high EZH2 expression was substantially linked with tumor stage, tumor size, histological differentiation, and lymph node metastasis^68^. Nevertheless, additional studies are required to investigate the role of this TF in CRC metastasis.

In the last part of this study, we investigated the DGIdb database for possible drugs targeting our final genes. In this regard, nine antineoplastic drugs were found. Among them, Bevacizumab, sold under the brand name Avastin is an approved drug for the treatment of metastatic CRC^69^. Lenalidomide and Plerixafor have also shown potential for metastatic CRC treatment. In a study by Galustian et al., they proved that Lenalidomide can inhibit metastatic CRC in vivo and in vitro^70^. On the other hand, Plerixafor has completed phase one trials for metastatic CRC^71^. Other drugs in the drug-DEGs network in this study have also found to be effective in the treatment of different metastatic cancers, such as breast cancer^72^ and thyroid carcinoma^73^. These drugs may have the potential to be repurposed as a treatment for metastatic CRC, providing new options for patients and physicians.

The mortality rate of cancer is closely linked to the stage of cancer progression, highlighting the possibility of reducing mortality through early detection and management^74^. This imperative is underscored by a substantial decrease in the 5-year survival rate, notably in instances of distant metastases, where the survival rate plummets to 10%^75,76^. The necessity to identify precise genes and pathways is increased aiming for timely diagnosis and personalized therapeutic strategies to effectively confront the intricacies of cancer progression and metastasis^77^. Importantly, the identification of stage-specific biomarkers in colorectal cancer is of great importance in this context, as it considerably enhances our ability for early detection and targeted interventions, thereby contributing significantly to addressing the challenges associated with colorectal cancer^78,79^.

The integration of ML algorithms into the analysis of existing datasets holds promising potential for identifying stage-specific biomarkers in colorectal cancer^80^. this advanced computational approach not only supports the necessity of early detection and intervention but also improves the accuracy and effectiveness of the biomarker selection process^81^. The application of machine learning, including algorithms like LASSO and P-SVM, introduces a nuanced and targeted methodology, augmenting our capability to discern key biomarkers associated with different stages of colorectal cancer^27,82,83^. This innovative approach represents an important step toward refining our understanding of cancer progression, establishing a foundation for the development of more effective diagnostic and therapeutic strategies tailored to specific stages of the disease^84^.

Although further investigations are needed, the present study contributes to a better understanding of CRC metastasis. It is crucial to consider the limitations of this study when interpreting its findings, including low liver metastases sample size due to the scarcity of liver metastasis samples of CRC from participating hospitals. This constraint may have affected the study's ability to detect statistically significant differences and limits the generalizability of the results.

## Conclusion

We employed two machine learning algorithms to identify biomarkers associated with CRC metastasis. Through these methods, a total of 11 biomarkers were identified, and four of them were experimentally validated. Also, the SVM model based on these 11 feature genes showed the optimal classification performance in identifying CRC liver metastasis samples. The joint application of these genes could be considered as a diagnostic panel for metastasis assessment, further augmented by the development of an innovative AI predictive model based on these genetic signatures. These findings make a significant contribution to the continuous pursuit of deeper comprehension regarding the intrinsic molecular mechanisms driving CRC metastasis, with potential implications for the advancement of more efficacious diagnostic and therapeutic strategies tailored to this affliction. Additionally, the use of machine learning approaches in this study highlights the potential of this method for identifying biomarkers in complex biological systems.

### Supplementary Information

Supplementary Table 1.

Supplementary Figure 1.

## Data Availability

The corresponding author can provide the datasets utilized in this study on a reasonable request. The datasets analyzed during this study are available in the GEO database (https://www.ncbi.nlm.nih.gov/geo/database with GSE41568, GSE41258 and GSE68468 accession numbers) and TCGA database (https://portal.gdc.cancer.gov/).

## Abbreviations

CRC: Colorectal cancer
DEG: Differentially expressed gene
mRNA: Messenger RNA
ML: Machine learning
DL: Deep learning
AI: Artificial intelligence
COAD: Colon adenocarcinoma
READ: Rectal adenocarcinoma
GO: Gene ontology
KEGG: Kyoto Encyclopedia of Genes and Genomes
GSEA: Gene set enrichment analysis
LASSO: Least absolute shrinkage and selection operator
SVM: Support vector machine
RF: Random Forest
ROC: Receiver operating characteristic
AUC: Area under ROC curve
