# Supplementary material for: Using machine learning approach for screening metastatic biomarkers in colorectal cancer and predictive modeling with experimental validation
Source: Sci Rep. 2023 Nov 8;13:19426. doi: 10.1038/s41598-023-46633-8 (PMC10632378; doi:10.1038/s41598-023-46633-8)
Supplement: Supplementary file 1 — Supplementary Table 1. [file 41598_2023_46633_MOESM1_ESM.docx]

| P-SVM | | LASSO | SCAD | RF | |
| --- | --- | --- | --- | --- | --- |
| GUCY1A2 | INHBE | TMEM158 | TMEM158 | WNT5A | FMO3 |
| MUC4 | TMEM158 | WNT5A | GPX3 | WNT11 | FGA |
| MMP1 | WNT5A | WNT11 | EPHA3 | FOXC1 | SFTA2 |
| FOXF1 | WNT11 | FOXC1 | PTGIS | GPX3 | GUCY1A2 |
| SLC2A3 | FOXC1 | GPX3 | FABP1 | PRELP | CPB2 |
| SLC2A14 | EPHA3 | EPHA3 | NKX2-3 | ITIH4 | DEPP1 |
| ISM1 | PRELP | PTGIS | TMEM158 | ORM2 | SELENBP1 |
| CXCL14 | TNFSF11 | FABP1 | GPX3 | ORM1 | MGP |
| VGLL3 | MMP3 | NKX2-3 | EPHA3 | AQP9 | PTGER4 |
| COL12A1 | MAP2 | TNFSF11 | PTGIS | FXYD1 | THBS2 |
| REEP6 | PDE4D | MMP3 | FABP1 | SERPINC1 | HMCN1 |
| EFEMP1 | CXCR4 | MAP2 | NKX2-3 | THRSP | F5 |
| CASP1 | MAB21L2 | PDE4D |  | ALB | PROM1 |
| MB | CEL | CXCR4 |  | GC | TRPA1 |
| FBLN1 | MICU3 | MAB21L2 |  | P2RX7 | FCGR2B |
| ELAPOR1 | PZP | RBP1 |  | ZC3H12C | COLEC11 |
|  |  | STEAP4 |  | LCN2 | SLITRK6 |
|  |  | FCN3 |  | ALB | TFPI |
|  |  | SMAD9 |  | IGFBP1 | FGG |
|  |  | CDH2 |  | FGB | HABP2 |
|  |  |  |  | BEST2 | ASPN |
|  |  |  |  |  | APOE |

Supplementary Table1. Genes selected by each algorithm
