# Supplementary material for: Using machine learning approach for screening metastatic biomarkers in colorectal cancer and predictive modeling with experimental validation
Source: Sci Rep. 2023 Nov 8;13:19426. doi: 10.1038/s41598-023-46633-8 (PMC10632378; doi:10.1038/s41598-023-46633-8)
Supplement: Supplementary file 2 — Supplementary Figure 1. [file 41598_2023_46633_MOESM2_ESM.docx]

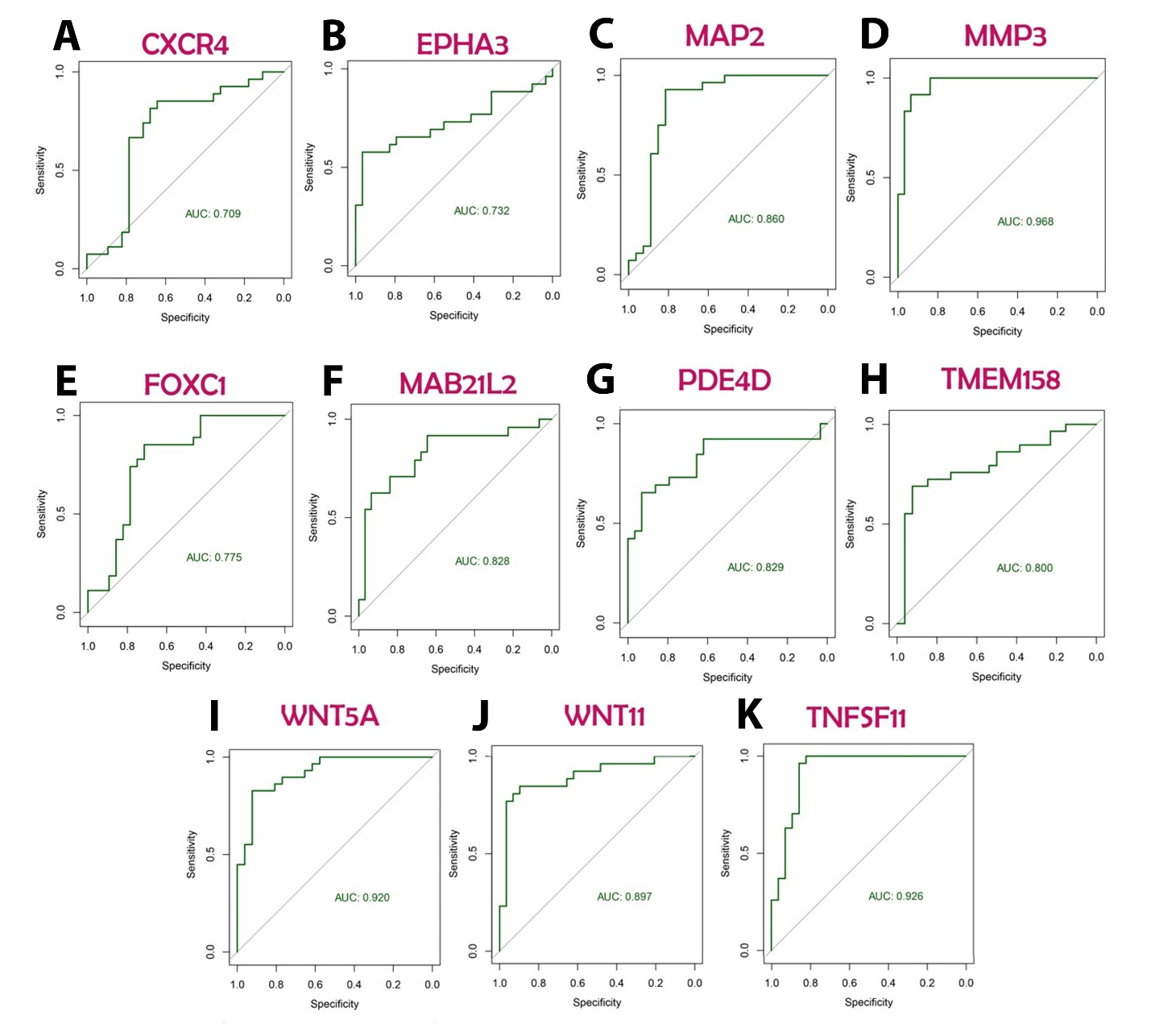


Supplementary Fig. 1. SVM method was applied to evaluate the efficiency of each gene in discriminating metastatic samples from non-metastatic ones. In this study, the area under the ROC curve (AUC)and the total accuracy were used to assess the models’ performances. ROC curves for feature genes including A) *CXCR4*, B) *EPHA3*, C) *MAP2*, D) *MMP3*, E) *FOXC1*, F) *MAB21L2*, G) *PDE4D*, H) *TMEM158*, I) *WNT5a*, J) *WNT11*, and, K) TNFSF11 is presented in this figure.
